# A Molecular Biophysical Approach to Diclofenac Topical Gastrointestinal Damage

**DOI:** 10.3390/ijms19113411

**Published:** 2018-10-31

**Authors:** Eduarda Fernandes, Telma B. Soares, Hugo Gonçalves, Sigrid Bernstorff, Maria Elisabete C. D. Real Oliveira, Carla M. Lopes, Marlene Lúcio

**Affiliations:** 1 CF-UM-UP, Centro de Física das Universidades do Minho e Porto, Departamento de Física da Universidade do Minho, Campus de Gualtar, 4710-057 Braga, Portugal; eduardabfer@gmail.com (E.F.); telmabsoares@gmail.com (T.B.S.); goncalves.hmc@fisica.uminho.pt (H.G.); beta@fisica.uminho.pt (M.E.C.D.R.O.); 2 Sincrotrone Trieste, SS 14 km 163.5, 34012 Basovizza, Italy; sigrid.bernstorff@elettra.eu; 3 FP-ENAS/CEBIMED—Fernando Pessoa Energy, Environment and Health Research Unit/Biomedical Research Centre, Faculty of Health Sciences, Fernando Pessoa University, 4249-004 Porto, Portugal

**Keywords:** diclofenac, NSAIDs, derivative spectrophotometry, steady-state and time-resolved fluorescence, steady-state anisotropy, DLS, DSC, SAXS, WAXS, gastrointestinal topical toxicity

## Abstract

Diclofenac (DCF), the most widely consumed non-steroidal anti-inflammatory drug (NSAID) worldwide, is associated with adverse typical effects, including gastrointestinal (GI) complications. The present study aims to better understand the topical toxicity induced by DCF using membrane models that mimic the physiological, biophysical, and chemical environments of GI mucosa segments. For this purpose, phospholipidic model systems that mimic the GI protective lining and lipid models of the inner mitochondrial membrane were used together with a wide set of techniques: derivative spectrophotometry to evaluate drug distribution at the membrane; steady-state and time-resolved fluorescence to predict drug location at the membrane; fluorescence anisotropy, differential scanning calorimetry (DSC), dynamic light scattering (DLS), and calcein leakage studies to evaluate the drug-induced disturbance on membrane microviscosity and permeability; and small- and wide-angle X-ray scattering studies (SAXS and WAXS, respectively), to evaluate the effects of DCF at the membrane structure. Results demonstrated that DCF interacts chemically with the phospholipids of the GI protective barrier in a pH-dependent manner and confirmed the DCF location at the lipid headgroup region, as well as DCF’s higher distribution at mitochondrial membrane contact points where the impairment of biophysical properties is consistent with the uncoupling effects reported for this drug.

## 1. Introduction

**Figure 1:**
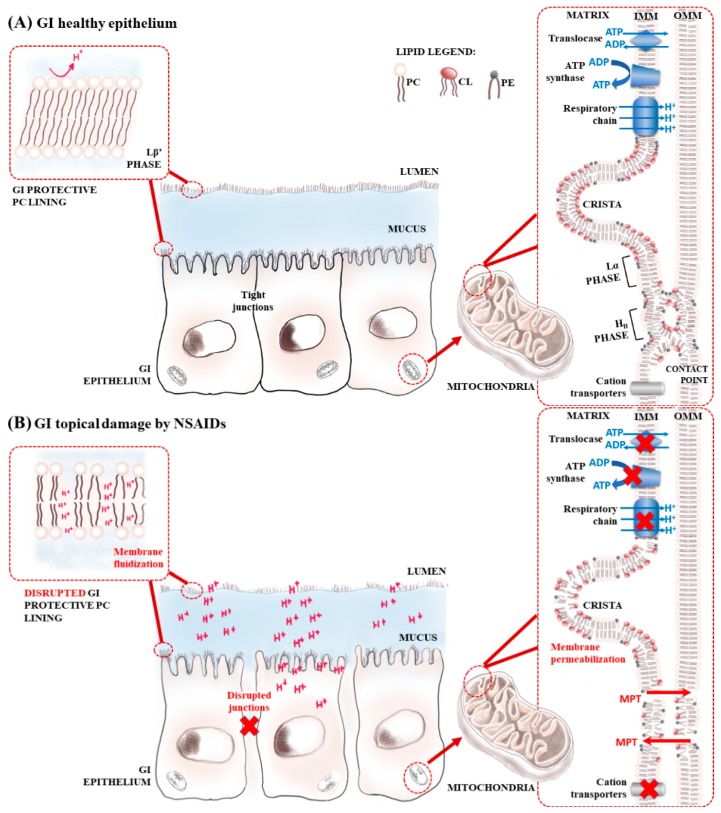
(**A**) Schematic representation of the gastrointestinal (GI) epithelium containing extracellular and cellular phospholipid linings rich in phosphatidylcholine (PC) in the gel phase (L_β′_), which constitute a protective barrier of the GI mucosa against the attack of luminal acid (H^+^). The mitochondria outer and inner membranes (OMM and IMM) containing typical crista and contact points rich in bilayer prone phospholipids (e.g., PC) that produce lamellar phases (L_α_) and non-lamellar prone lipids (e.g., Cardiolipin (CL) and phosphatidylethanolamine (PE)) that produce inverted hexagonal phases (H_II_) are also represented. (**B**) Schematic representation of GI topical damage depicting the disruptive effect of non-steroidal anti-inflammatory drugs (NSAIDs) on the GI protective phospholipid lining. On the right are represented the possible mechanisms by which NSAIDs cause mitochondrial toxicity: inhibition of respiratory chain; inhibition of ATP synthase and translocase; blockage of cation transporters; and membrane permeabilization with the formation of membrane permeability transition pores (MPT).

Diclofenac (sodium 2-[2-(2,6-dichloroaniline)phenyl]acetate) (DCF) is a non-steroidal anti-inflammatory drug (NSAID) generally used clinically, having an estimated global annual consumption of about 940 tons, including for human and veterinary purposes [1]. Despite its vast clinical use, DCF presents typical NSAID-related adverse effects of which gastrointestinal (GI) complications (e.g., GI bleeding and gastric ulceration) are the most relevant [2,3,4,5]. These GI effects have been considered to result mainly from the inhibition of cyclooxygenase (COX) enzyme, causing a decrease of prostaglandins that exert a strong protective effect on gastric mucosa, a mechanism known as prostaglandin hypothesis [6]. However, it is now recognized that the induction of GI lesions involves additional mechanisms (named the topical effect hypothesis), which are dependent on NSAIDs’ chemical structure and on their molecular ability to interact with lipids resulting in the damage of GI mucosa and cellular mitochondria impairment [6,7,8,9,10] (Figure 1). Moreover, besides GI injuries, DCF and chemical structure-related NSAIDs present, in susceptible patients, additional deleterious effects such as hepatic, renal and cardiovascular damage that cannot be explained by the prostaglandin hypothesis mechanism, but are instead associated with oxidative stress and topical effects such as mitochondrial liabilities [6,11,12,13,14,15,16,17,18,19,20].

The topical effect of NSAIDs and their role in causing GI damage can be explained by a biophysical interaction between drug molecules and phospholipids. GI cells are protected by a mucus layer that serves as a matrix of phospholipids of which phosphatidylcholines represent a prominent component. The phospholipid lining provides a resistant gel layer with a non-wettable hydrophobic surface that protects the epithelium from luminal acid [7,8,9,10,21,22]. NSAIDs interact with the phospholipid lining of the GI mucus layer and the cell surface phospholipid bilayer, compromising their biophysical integrity. This increases the mucus layer wettability and leads to GI mucosal exposure to luminal acid and other aggressive agents (pepsin, bacteria and bile) with the consequent development of erosions [6,21] (Figure 1). Although several studies have been conducted to evaluate the role of COX selectivity in GI toxicity of NSAIDs, less attention has been paid to the biophysical effects of the NSAIDs-membrane interaction, knowledge of which is essential to understand these drugs’ GI topical effect [6,8,9,10,21]. Only recently, studies have focused on the biophysical factors that may account for the apparent low ulcerogenicity of some NSAIDs in comparison with others that demonstrate higher toxicity. Studies involving membrane model systems mimicking the phospholipid lining of the GI protection barrier have reported fluidizing and disordering effects of NSAIDs, indicative of their GI toxicity. This was the case with indomethacin, acemetacin, piroxicam, tolmetin, celecoxib, aspirin, naproxen and ibuprofen [10,23,24,25,26,27,28,29,30], which were all able to destabilize the gel phase of the GI mimetic phospholipid lining. By contrast, nimesulide and etoricoxib, which are safer at the GI level, revealed the least perturbing effects on the lipid gel phase and on the lipids extracted from the GI brush border membrane, respectively [23,30,31].

Furthermore, NSAIDs also demonstrated a capacity to interfere with the mitochondrial function through several pathways as an additional role contributing to NSAID’s GI toxicity (Figure 1). NSAIDs’ uncoupling effect of oxidative phosphorylation or inhibition of electron transport in mitochondria causes a reduction of adenosine triphosphate (ATP) cellular levels, damaging the intercellular junctions in the GI tract, increasing mucosal permeability, and ultimately causing apoptosis and GI cell death [6,11]. In this regard studies performed in isolated mitochondria reported that DCF can act as an uncoupler agent of mitochondrial oxidative phosphorylation and can instigate the aperture of a channel-type structure in the inner mitochondrial membrane called the mitochondrial permeability transition pore (MPT).

MPT results in the dissipation of the proton gradient across the inner mitochondrial membrane and, consequently, in the collapse of the transmembranar potential (i.e., membrane depolarization) and in the decrease of ATP synthesis [18,19,32,33]. DCF can inhibit mitochondrial ATP synthesis at a therapeutic relevant concentration [18,34] and some authors demonstrated time and dose dependent effects of DCF in the capacity to perturb mitochondrial function [18,35,36]. Additionally, DCF interacts with several ion channels from different cells, including voltage-gated Na^+^, Ca^2+^ channels, ligand-gated K^+^, transient receptor potential (TRP), and other cation nonselective channels affecting the ion homeostasis in the mitochondrial membrane [37]. Related to this effect, a more pronounced mitochondrial swelling (i.e., MPT) of DCF has been demonstrated in the presence of Ca^2+^ [19].

Although disruption of the mitochondrial function is recognized as a common occurrence among the adverse effects of DCF, its in vivo mechanism is not yet fully understood [38] and studies concerning the biophysical topical effect of DCF at the mitochondrial membrane level are required. Indeed, the mitochondrial bioenergetic function can also be impaired by membrane biophysical changes that may affect cardiolipin (CL), thereby inhibiting specific transporters in the mitochondrial membrane [36]. CL is a signature phospholipid of the inner mitochondrial membrane (IMM) capable of forming lamellar and non-lamellar lipid structures. This polymorphic capability is essential to the normal functioning of the IMM so drug-induced changes in this ability are possibly harmful to the normal functioning of membrane bound proteins [39,40,41,42].

Some studies have been performed to investigate the molecular interactions between DCF and lipid membranes [25,43,44,45]. The results of these studies suggest the facility of DCF to penetrate and interact with lipid membrane environments and, hence, its ability to modify membrane biophysical properties with impacts on physiological membrane-related functions. The studies reported are very relevant for a broad understanding of DCF–membrane interactions; however, lipophilicity, acid–base properties, the lipid phase, and the composition of the membrane models used play a determinant role in the drug-membrane interactions [24,46,47]. A more specific biophysical approach contemplating these parameters to study DCF effects on phosphatidylcholine-rich GI linings is missing in the literature. Furthermore, previously reported in vitro studies performed in rat and human cellular mitochondria have proved toxicity induced by DCF and suggested that DCF’s functional groups (probably carboxylic acid) might play a fundamental role in such effects [18,36,48]. Nevertheless, to our knowledge, there are no biophysical studies concerning the interaction of DCF with CL-rich membrane models that can confirm this assumption and reinforce the topical aspect of DCF mitochondrial toxicity.

In summary, the main aim of this work is to contribute to a better understanding of DCF’s GI topical toxicity using relevant pH conditions and membrane models that mimic the biophysical and chemical environment encountered by the drug: (i) in the phospholipid lining protective barrier of GI mucosa; and (ii) in the IMM. Several biophysical techniques were used to provide a molecular, thorough study: derivative spectrophotometry to evaluate DCF’s distribution at the membrane; steady-state and time-resolved fluorescence to predict DCF’s location at the membrane; fluorescence anisotropy, differential scanning calorimetry (DSC) and dynamic light scattering (DLS) to assess DCF’s effect at membrane microviscosity; calcein leakage studies to study DCF’s effects on membrane permeability; and synchrotron small- and wide-angle X-ray scattering studies (SAXS and WAXS, respectively), to evaluate the effects of DCF at the membrane structure. The gathered results confirm DCF’s effects on the impairment of membrane biophysical properties, which are in line with the reported effects on membrane fluidity, transmembrane potential, and protonophoric activity, and have an impact on physiological membrane-related functions and on DCF’s local gastric toxicity.

## 2. Results

### 2.1. Diclofenac Distribution Coefficient in a Membrane/Aqueous System

**Figure 2:**
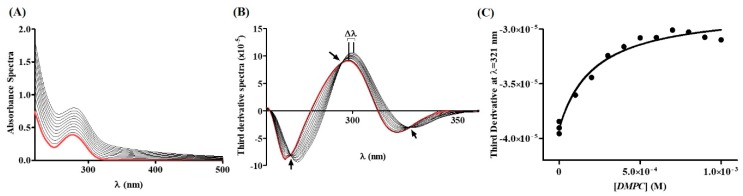
(**A**) DCF (40 μM) absorption spectra in aqueous buffered phase pH = 5.0 (red spectrum) and after incubation with increasing concentrations of LUVs of DMPC (0–1000 μM) prepared in the same buffer pH = 5.0 (black spectra); (**B**) third derivative of the absorbance spectra where it is possible to observe isosbestic points (arrows) and a shift of λ values at the peaks of the derivative spectra (Δλ); and (**C**) nonlinear fitting of derivative absorbance values at λ = 321 nm as a function of lipid concentration (DMPC).

The first physicochemical property of NSAIDs that provides solid evidence to support the distribution of these drugs within the PC of the GI protective layer is the distribution coefficient (*K*_d_) between lipid/aqueous phases, which allows evaluation of the drug’s distribution in the hydrophobic and hydrophilic microenvironments [46]. Accordingly, the *K*_d_ of DCF was determined in LUVs (large unilamellar vesicles) of 1,2-dimyristoyl-*sn*-glycero-3-phosphocholine (DMPC) at pH 5.0 and at 37 °C by derivative spectrophotometry. The membrane model system was chosen to mimic the PC lining present in both extracellular and membrane barriers (Figure 1) [6,10]. The studies were performed at acidic pH of 5.0 to mimic the average pH values encountered by these drugs in the upper parts of the GI where drug absorption occurs [46]. The pH value of 5.0 was chosen based on the posologic regimen of NSAIDs that recommends administration after meals [49]. Thus, this is the value reported to be the pH of luminal gastric and upper intestinal segments in a fed state [46]. Examples of the absorbance spectra and subsequent derivative method for data analysis are presented in Figure 2.

From the absorbance spectra of DCF experimentally obtained for increasing concentrations of LUVs of DMPC (Figure 2A), the third derivative spectra were calculated (Figure 2B), as this eliminates the effect of Rayleigh dispersion, which is higher for smaller wavelengths [50]. Third derivative spectra analysis eliminates light-scattering interference and improves spectral resolution, enhancing details of DCF–lipid interaction. Indeed, at the derivative peaks, it is possible to observe a shift of λ (of about 10 nm; see Table 1), indicative of DCF distribution into environments of lower polarity (distribution of DCF into the lipid phase) [51] with a decrease in the intensity of the bands as the lipid concentration increases. Isosbestic points are also observed, indicating the existence of a balance between two DCF forms (interacting with the lipid medium and free in aqueous buffered medium) and the eradication of light-scattering interference [50]. Using maximum or minimum values from derivative spectra (e.g., 321 nm), data were plotted against the respective DMPC concentration (Figure 2C) and the resulting data points were fitted to Equation (1) (Figure 2C, black line). From that fitting, *K*_d_ values were calculated (Table 1).

For comparison, in Table 1 are presented the reported *K*_d_ values in biphasic membrane/aqueous systems at two other pH values: 3.0 and 7.4 [43]. The values are dimensionless and resulted from the correction of the lipid molar volume of each model system so that they can be compared.

The *K*_d_ of DCF into PC membranes was shown to be deeply dependent on the pH values. This different distribution of DCF should be attributed to the different ionization state of the drug and reflects the importance of studying NSAID-lipid interaction using lipid model systems at different relevant physiological pH values. The pKa of DCF has been reported to be 3.97 ± 0.04; thus, at pH 3.0 (pH < pKa), the drug molecule is in the neutral protonated form and the distribution within the PC membranes is maximum (Log *K*_d_ = 4.41 ± 0.04) [43]. At pH values higher than the pKa (pH 5.0 and 7.4), the drug distribution decreases as the pH increases. This is due to the presence of negatively charged ionic forms of DCF, which, because they are more polar, are less distributed in the lipid media than the neutral forms. Furthermore, according to the Henderson-Hasselbach equation, at pH 7.4, the concentration of negatively charged DCF is 2692 times higher than the concentration of the drug’s neutral form (which is almost nonexistent), whereas, at pH 5.0, the concentration of the negatively charged DCF is 10 times higher than the concentration of the drug’s neutral form. These differences confirm that the DCF neutral form is more distributed within PC membranes as well as indicate that DCF can be distributed within PC membranes even at pH 7.4, when its ionization is practically 100%. In this latter case, drug distribution within lipid membranes should also be ruled by electrostatic interactions established between negatively charged carboxyl groups of DCF and PC’s positively charged choline groups.

### 2.2. Diclofenac Location in a Membrane/Aqueous System

After quantifying DCF distribution between the lipid and aqueous phases of the membrane model system, it is important to assess where the drug will be most likely located within the lipid phase of the membrane. Assessing drug location at mimetic systems of the GI protective layer will allow us to understand if the drug is more superficially bounded in the PC headgroups or if it is more deeply buried at the hydrophobic microenvironment of the PC acyl chains.

**Figure 3:**
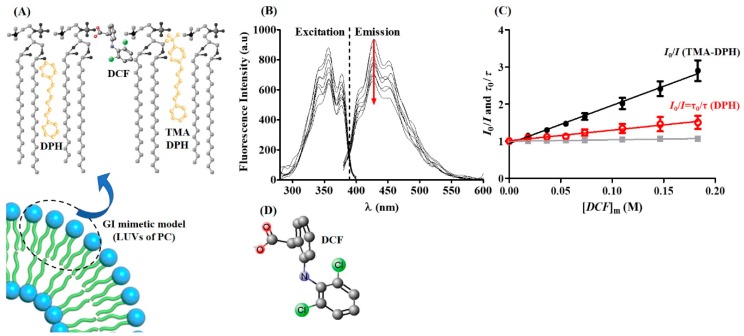
(**A**) Schematic representation of the possible location/orientation of the fluorescent probes TMA-DPH and DPH (yellow structures) and the drug DCF within PC molecules of the lipid bilayer. (**B**) Excitation and emission spectra of the fluorescence probe TMA-DPH incorporated in LUVs of DMPC. Red arrow shows the fluorescence quenching effect of increasing concentrations of DCF added to the labeled system at a temperature of 37 °C and pH 5.0. (**C**) Stern-Volmer plots of the probes TMA-DPH and DPH incorporated in LUVs of DMPC at a temperature of 37 °C and pH 5.0 as a function of DCF membrane concentrations. Grey symbols (■) were obtained from fluorescence lifetime measurements of TMA-DPH; black closed symbols (●) were obtained from fluorescence steady-state measurements of TMA-DPH; and red open symbols (◯) were obtained from fluorescence steady-state/lifetime measurements of DPH. All are average values ± standard deviation of at least three independent measurements. Lines are the best fit according to Equation (2). (**D**) DCF molecule with the quenching moieties (O, N, and Cl) highlighted.

For this purpose, it is possible to label the lipid model system with fluorescent extrinsic fluorophores, such as *N,N,N*-Trimethyl-4-(6-phenyl-1,3,5-hexatrien-1-yl)phenylammonium *p*-toluenesulfonate (TMA-DPH) and 1,6-Diphenyl-1,3,5-hexatriene (DPH) fluorescent probes. When incorporated in the membrane, the position of these probes is known, and they work as molecular rulers capable of labeling different depths of the lipid bilayer (Figure 3A). TMA-DPH possesses a cationic group that anchors the probe at the polar headgroup region of the membranes being described as a reporter of more ordered shallow regions of the bilayer (glycerol backbone and upper segments of the phospholipid acyl chains) while DPH is used to report deeper regions of the phospholipid acyl chains [52,53,54].

In this regard, TMA-DPH- and DPH-labeled PC model systems (LUVs of DMPC) used to mimic the PC-enriched GI lining were used to incorporate increasing concentrations of DCF at different temperatures and at pH 5.0. For both the labeled systems and for all the temperatures tested, a decrease in the intensities of fluorescence excitation and emission (fluorescence quenching) was observed as illustrated for TMA-DPH at 37 °C (Figure 3B). Additionally, the fluorescence quenching effect induced by DCF was concentration dependent. The quenching efficiency of DCF can be better evaluated by the observation of the correspondent Stern-Volmer plots obtained by fitting measurement data to Equation (2). Figure 3C shows, as an example, the Stern-Volmer plots obtained for fluorescence quenching of the probe TMA-DPH and DPH induced by increasing DCF concentrations and determined by steady-state and lifetime measurements at 37 °C. From the Stern-Volmer plots and using Equation (2), it was possible to determine the Stern-Volmer constant values (*K*_SV_) for both probes at several temperatures (Supplementary Table S1) thereby defining the type of quenching mechanism that was further confirmed by lifetime measurements. In the case of the static quenching mechanism, lifetime values did not change and *τ*_0_/*τ* = 1, as observed in Figure 3C for TMA-DPH. Accordingly, it was possible to conclude that DCF induced a fluorescence quenching of TMA-DPH by a static mechanism while DPH was quenched by the drug by a collisional mechanism.

Although *K*_SV_ values are used to evaluate the extension of the quenching for each probe individually, the comparison of quenching effects using the two different probes (TMA-DPH and DPH) requires the calculation of the bimolecular quenching rate constant (*K*_q_) that indicates the accessibility of the probes to the quencher (DCF), thereby eliminating specificities related with differences in probes’ fluorescence lifetime in the absence of the quencher. The *K*_q_ constants resultant from DCF quenching of TMA-DPH and DPH fluorescence when the probes were incorporated in a PC mimetic GI model were calculated for two lipid membrane phases (Table 2). The lamellar gel phase (L_β′_) is relevant to mimic the gel-ordered phase of the GI phospholipid lining, while the lamellar fluid phase (L_α_) is considered the most predominant phase in cellular membranes and can also mimic disordered domains on a damaged GI phospholipid lining [7,9,10].

The analysis of *K*_q_ values obtained in the GI mimetic model indicates that: (i) DCF can quench the fluorescence of both probes; (ii) in both lipid phases, *K*_q_ values are significantly higher for the TMA-DPH probe; and (iii) for the probe TMA-DPH, the *K*_q_ values are higher in the L_β′_ phase in contrast to those observed for the probe DPH. These results allowed us to propose that DCF orientation is parallel to the phospholipid molecules with the ionized negatively charged carboxyl group anchored at the polar PC headgroup region (Figure 3A). The presence of three quencher moieties in the DCF molecule (Figure 3D) justifies that at this proposed orientation the drug is accessible and quenches both probes. However, DCF ionization at pH 5.0 dictates its anchoring to the polar regions of the lipid membrane, which is reflected in higher quenching efficiencies of TMA-DPH. The higher accessibility to TMA-DPH is further confirmed by the static quenching mechanism observed for this probe, which fits well with our predicted location (Figure 3A). According to this prediction, the dichlorophenyl group of DCF may establish a stacking interaction with the phenyl group of TMA-DPH, which, by being also anchored to the polar headgroup region, has a shallower location than DPH (Figure 3A). Indeed, this type of chemical-stacking interaction has been described as responsible for static quenching mechanisms [55]. It is also relevant to say that the L_β′_ phase, in possessing better lipid packing, may constitute a hindering factor to the drug accessibility to the probes. Nonetheless, for the TMA-DPH probe, the *K*_q_ values were higher in the L_β’_ phase (Table 2), highlighting the capacity of DCF to penetrate the phospholipid gel lining protective of the GI tract and corroborating the drug’s potential topical GI toxicity.

As described for other NSAIDs, after DCF penetration in the phospholipid gel lining of the GI tract, the drug can diffuse across gastric mucosal epithelial cell membranes into the cytoplasm, where pH is neutral and where it finds another target of NSAIDs’ topical effect: mitochondria [6,11,56]. NSAIDs’ uncoupling effects at the mitochondria result from multiple pathways, of which the least explored is their molecular biophysical interaction with the mitochondria membrane [57,58]. In the case of DCF, there are no studies concerning this approach and thus we propose using an IMM mimetic model to investigate how DCF distributes at the mitochondrial-enriched CL membranes. To also appraise DCF distribution in lipid phases characteristic of IMM, quenching effects were evaluated when the former fluorescent probes were incorporated in liposomes composed of a mixture of DOPC:DOPE:CL (1:1:1). Quenching assays were performed at pH 7.4 characteristic of cytosolic mitochondria and at different temperatures at which the lamellar (L_α_) or inverted hexagonal (H_II_) arrangement of lipids prevailed [58,59,60]. These different lipid phases mimic the two morphological hallmarks of mitochondria, namely cristae of the IMM (rich in CL lamellar fluid lipid structures) and contact sites between the IMM and the outer mitochondrial membrane (OMM) that are likely to involve nonlamellar CL enriched lipid structures [40]. Once more, in this lipid model system, and at any of the lipid phases, DCF was able to quench both probes; the resultant *K*_q_ values are presented in Table 2. In this case, the analysis of *K*_q_ values indicates that the quenching is higher for the TMA-DPH probe, reflecting, as previously observed, a preferential orientation of DCF molecules anchored to the polar headgroup regions of the lipid arrangements. Moreover, DCF has higher quencher efficiencies in conditions at which the mixture of lipids is preferentially organized in the H_II_ phase. In fact, at this phase and for both probes, *K*_q_ values are about 40% higher. These findings suggest a preferential distribution of DCF through the H_II_ contact sites of mitochondria membrane. Once the nonlamellar arrangements are reported as essential for normal functioning of mitochondria, the preferential distribution of DCF within these nonlamellar arrangements may be potentially detrimental to other mitochondrial membrane-bound structures [40] and might be related to the MPT effects reported for this drug.

### 2.3. Effects of Diclofenac in the Microviscosity and Permeability of the Membrane

**Figure 4:**
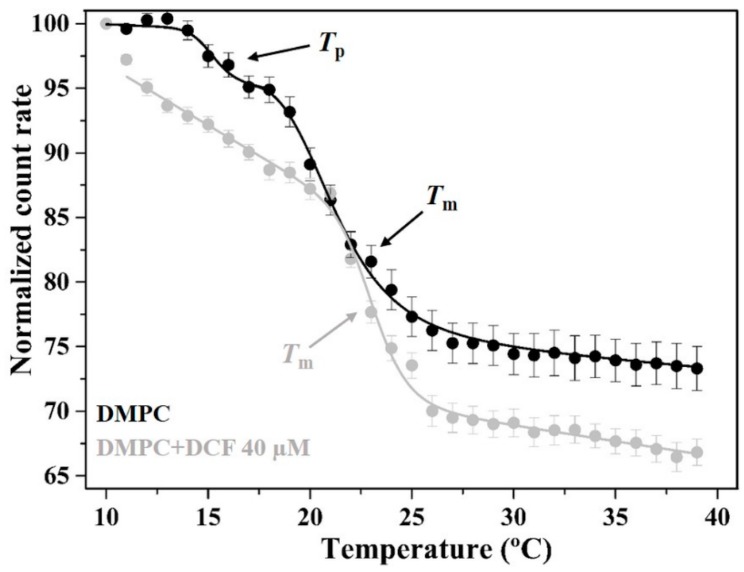
Normalized count rate of DMPC lipid vesicles unloaded and DCF-loaded (40 µM) as a function of temperature. Continuous lines are the best-fitted curves according to Equation (8). Values reported are the mean ± standard deviation of three replicate samples. *T*_p_ and *T*_m_ stand for pre-transition temperature and main phase transition temperature, respectively.

Besides studying how DCF distributes within a membrane model mimetic of GI protective lining, it is also important to study how the drug can affect the biophysical integrity of such lining with consequent exposure of mucosa cells to aggressive agents, resulting in ulcer formation and bleeding [10]. Evidence has supported the concept of NSAID-induced perturbation of membrane biophysical properties (e.g., by decreasing membrane microviscosity or by altering lipid structure and packing) translating into an increase in the permeability of biological membranes, sometimes with fusion effects [10,21,23,25,27,28,45,57,61]. In this context, the effects of DCF in the thermotropic behavior of PC model systems (LUVs of DMPC) used to mimic the PC-enriched GI lining were evaluated at pH 5.0 by DLS and DSC. Evaluation of the thermotropic behavior of lipid membranes by DLS is based on the fact that the measured count rate (average number of photons scattered by the liposomes analyzed detected per second) reflects changes in the optical properties of lipid vesicles with temperature variations. Accordingly, lipid systems in more ordered phases induce higher light scattering and lead to higher count rate values than the fluid lipid systems [29,62]. The count rate of DMPC liposomes measured as a function of temperature in the absence and presence of DCF at pH 5.0 is shown in Figure 4.

In the absence of the drug, DMPC lipid vesicles presented a high cooperativity (248 ± 32), suggesting that the lipid molecules transit to a different phase practically at the same time. Furthermore, it was possible to notice the presence of typical transitions: (i) pre-transition temperature (*T*_p_ = 15.4 ± 0.4) from the L_β’_ to the ripple phase (P_β’_); and (ii) main phase transition temperature (*T*_m_ = 22.8 ± 0.2) from the P_β’_ to the L_α_ phase. When DCF is added to the lipid system, it abolishes the *T*_p_ and does not affect *T*_m_ but reduces the cooperativity of the main phase transition. The abolishment of *T*_p_ indicates interactions established between DCF and the polar headgroups of PC membranes [63] and corroborates the shallow location of this NSAID predicted by fluorescence quenching. The observed decrease of the cooperativity of the main transition is also in agreement with the DCF location within the polar headgroup region since the cooperative unit that undergoes the transition is largely regulated by the interaction in the carbons C_1_–C_8_ of the acyl chains [64]. Moreover, despite not changing *T*_m_, DCF has a significant effect in reducing DMPC membrane microviscosity in the gel and fluid phases, as observed by the significant decrease of count rate values in both phases. This indicates that, in the presence of DCF, the lipid bilayer system becomes more fluid and thus less capable of scattering light. DSC measurements confirmed the overall conclusions obtained with DLS (Supplementary Figure S1). From DSC thermogram, it was possible to conclude that DCF abolished the pre-transition peak and reduced the cooperativity of the phase transition without significantly changing enthalpy. These observations reinforce that the predicted drug location is closer to the aqueous lipid interface. However, with this technique it was possible to observe a slight reduction (0.34 °C) of *T*_m_ when DCF was incorporated in the lipid system. Although to a smaller extent, this observation agrees with previous DSC studies that account for a DCF-induced disordering effect at DMPC membranes [25]. Indeed, Manrique-Moreno and co-workers observed that the addition of increasing concentrations of DCF to the lipid system induced the disappearance of the ripple phase and a shift of *T*_m_ for lower temperatures. The extent of this shift was about 2 °C for a lipid:drug molar ratio of 1:0.1 at pH 7.4 [25]. It should be noted that we have tested a significantly lower lipid:drug molar ratio (1:0.03) to observe an effect at DCF concentrations closer to therapeutic levels, and we have studied the drug–lipid interaction at pH 5.0 to mimic the pH of the PC lining of the luminal gastric and upper intestinal segments in a fed state.

**Figure 5:**
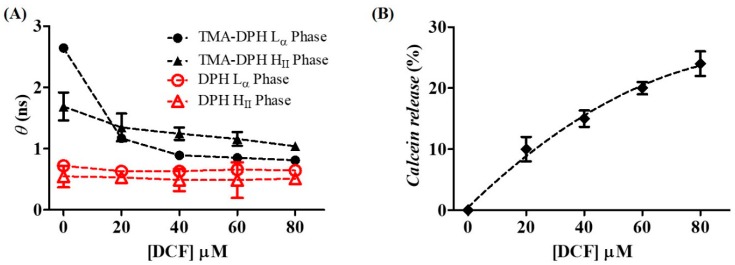
(**A**) Rotational correlation time (*θ*) of TMA-DPH (closed black symbols) and DPH (open red symbols) in DOPC:DOPE:CL (1:1:1) liposomes at L_α_ phase (circles) and H_II_ phase (triangles) as a function of DCF concentration. (**B**) Calcein release from DOPC:DOPE:CL (1:1:1) liposomes at L_α_ phase as a function of DCF concentration. Calcein release (%) was calculated according to Equation (7). Results are representative of at least three independent determinations ± standard deviation. Dashed lines are guidelines only.

To study how DCF can affect the lipid dynamics and phospholipid acyl-chain packing of the mitochondrial membranes, fluorescence anisotropy and calcein release studies were performed by the addition of increasing drug concentrations to an IMM mimetic model made of liposomes composed of a mixture of DOPC:DOPE:CL (1:1:1) at pH 7.4. In the case of steady-state anisotropy measurements, the rotational correlation time (*θ*) was calculated for the same fluorophores used in fluorescence quenching studies (TMA-DPH and DPH), incorporated in the IMM mimetic model at different temperatures at which the L_α_ or H_II_ arrangement of lipids prevailed (Figure 5A).

Since *θ* gives information about the probe motion, it will report the fluidity of the surrounding environment of the probe. Therefore, if DCF added to the lipid model system affects the membrane fluidity, it can alter the probe motion, producing changes in *θ* values. Accordingly, as shown in Figure 5A, it is possible to conclude that DCF promotes a significant fluidizing effect at the polar regions of the L_α_ phases characteristic of IMM cristae. However, to a lesser extent, DCF also promotes a fluidizing effect at the polar headgroup regions of the H_II_ phases characteristic of contact sites between IMM and OMM. However, at the inner regions of the L_α_ and H_II_ phases, DCF has a negligible effect on hydrocarbon chain packing. This was expected given the preferential distribution of DCF in the headgroup region confirmed by fluorescence quenching, DSC, and DLS studies.

Another way of studying if DCF affects the biophysical integrity of the mitochondrial membrane is the study of membrane permeability by calcein release. Liposomes of DOPC:DOPE:CL (1:1:1) simulating the L_α_ phase of the IMM were loaded with calcein at a concentration where self-quenching of this probe occurs. After addition of DCF to the membrane mimetic system, membrane permeabilization was followed by calcein release, measuring the fluorescence intensity increase. As shown in Figure 5B, DCF increased the permeability of the membrane system assayed in a dose-dependent manner, reaching 25% of calcein leakage at the maximal concentrations tested.

### 2.4. Effects of Diclofenac in Lipid Packing and Membrane Structure

Membrane fluidity is a complex biophysical aspect of biomembranes that encompasses a combination of dynamic and orientational properties of the constituents of membrane lipid bilayer. When studying the effect of drugs on the fluidity of membrane lipid model systems, it is therefore necessary to analyze different properties, such as membrane microviscosity and permeability, as well as membrane structure and lipid packing parameters [65,66]. In this regard, the effects of DCF in different DPPC (1,2-dipalmitoyl-*sn*-glycero-3-phosphocholine) lipid phases were evaluated by SAXS and WAXS diffraction patterns providing information on the bilayer structure and lipid packing through the respective measurement of long and short spacings (*d* values). The studies were conducted in a DPPC lipid model at pH 5.0 to mimic the PC content of the phospholipid GI lining, particularly given palmitoyl derivatives are associated with the integrity of the protective GI barrier. The studies were performed in temperatures ranging from 20 to 50 °C to comprehend the different lipid phases; however, special attention has been paid to the L_β’_ representative of the GI phospholipid lining [7,9,10].

**Figure 6:**
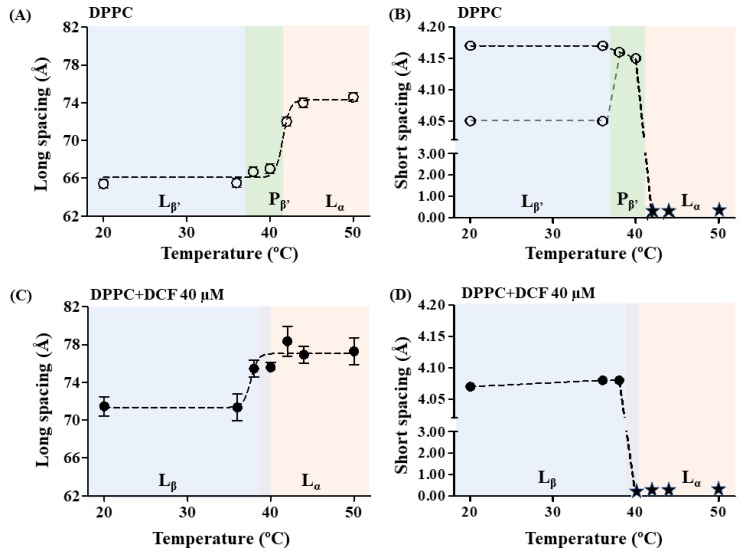
Long and short spacing obtained, respectively, by SAXS (**A**,**C**) and WAXS (**B**,**D**) measurements of DPPC multilayers made at several temperatures and at pH 5.0 in the absence (open symbols) and in the presence (closed symbols) of DCF. Values are presented as average spacing ± standard deviation calculated from all diffraction peaks. Dashed lines are guidelines only, and star symbols are temperatures at which no Bragg peaks appear in the WAXS region. L_β,_ L_β’_, P_β’_, and L_α_ stand for lamellar gel phase (β stands for untilted and β’ stands for tilted), ripple phase, and lamellar fluid phase, respectively.

Typical SAXS and WAXS patterns were obtained for L_β’_, P_β’_, and L_α_ phases of fully hydrated DPPC at pH 5.0 in good agreement with the literature [26]. From the SAXS patterns of DPPC, the lamellar long spacing was determined in each lipid phase (Supplementary Table S2) and the long spacing values are represented as a function of temperature in Figure 6A.

In the L_β’_ phase, the bilayer thickness, including a water layer between the bilayers, was determined to be 65.4 ± 0.5 Å (see also Supplementary Table S2), similar to values that have been reported in the literature [26]. On heating, the spacing increases to 67.0 ± 0.5 Å in the P_β’_ phase and to 74.0 ± 0.5 Å in the L_α_ phase (Supplementary Table S2). The profile obtained for the long-spacing values as a function of temperature (Figure 6A) indicates a transition to the L_α_ phase at ca. 42 °C, in agreement with the main phase transition found for DPPC [23,26,67]. Deconvolution of the WAXS patterns obtained at the L_β’_ phase gives the lattice constants of two peaks characteristic of an orthorhombic lattice with tilted chains, i.e., short spacing values of 4.05 ± 0.05 Å and 4.17 ± 0.05 Å (Figure 6B and Supplementary Table S2), which are also in good agreement with the results described for DPPC lipid systems at pH = 5.0 [26]. Increasing temperature leads to a decrease of the lattice distortion, and at ripple phase, the two Bragg peaks of the orthorhombic lattice come closer together. At temperatures ≥42 °C, the WAXS peaks were too broad to be assigned, confirming the main phase transition found for the lipid system in the SAXS measurements and literature [23,26,67].

The diffraction peaks of the DPPC system in the absence of the drug present a small full width of the peaks at one-half of their intensity (*fwhm*), indicating a good correlation between the bilayers (see values of correlation length, *ξ*, in Supplementary Table S2). Such a correlation is profoundly reduced by the addition of DCF to the lipid system, manifesting the disturbance effect of this NSAID on the membrane structure (Supplementary Table S2). The broadening of the characteristic DPPC Bragg reflections in the case of the DCF loaded membrane model systems (Supplementary Figure S2) indicates the breakdown of the multilamellar correlation due to the inclusion of the drug [65]. The profile of the long-spacing values as a function of temperature obtained for DPPC systems containing DCF (Figure 6C) indicates a main transition temperature occurring at ca. 40 °C, confirmed by the short-spacing values as a function of temperature (Figure 6D). Accordingly, at temperatures ≥40 °C, the WAXS peaks were too broad to be assigned, confirming the presence of a L_α_ phase.

Figure 6C also shows an increase in the thickness of the bilayer plus the aqueous layer at the entire range of temperatures assayed in comparison to the same profile obtained for the lipid membrane system without the drug (Figure 6A). At temperatures corresponding to the more disordered phase (L_α_ fluid phase), this increase of the long-spacing values was 2.3 ± 0.2 Å, which may be attributed to an increase in the hydration layer. Indeed, the location of the drug in the polar headgroups can modify the surface-bond water molecules in the bilayer, as previously stated to happen with DCF and other NSAIDs in a DSC study made in DMPC model systems at physiological pH [25].

**Figure 7:**
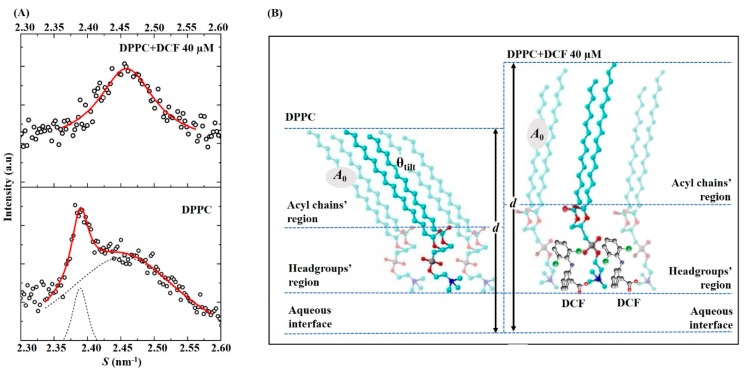
(**A**) WAXS patterns recorded in static exposures at 20 °C and at pH 5.0 for multilayers of DPPC in the absence (lower figure) and presence of DCF. Solid red lines give the best fit of the Lorentzian’s analysis model to the scattered intensities. (**B**) Schematic representation of the effect of DCF penetrating the headgroup region of DPPC causing the loss of tilt angle with consequent increase of the long spacing (*d*) and decrease of cross-sectional area (*A*_0_) of the aliphatic lipid chain.

At temperatures corresponding to the more ordered phases of DPPC (gel and ripple phase), an average increase in the long spacing of 7.3 ± 1.6 Å was observed upon DCF addition in comparison to that observed for the lipid membrane system without the drug. The increase of *d* values at the lipid ordered phases should be also caused by the interaction of DCF with the polar headgroups of the lipid membrane. Such interactions could reduce the effective area requirement of the PC headgroups by two possible effects: (i) reduction of the chain’s tilt angle; and/or (ii) increasing of the water layer. We can thus start to analyze if this increase in the *d* values is coherent with a tilt angle change by some simple deductions. In the gel phase, the hydrocarbon chains of the fatty acids constituting the phospholipids are rigidly packaged, with their C–C bonds in an *all-trans* conformation, which allows the accommodation of the hydrophobic chains in a minimum volume. These *all-trans* C–C bonds organized in CH_2_ groups in hydrocarbon chains are at an interval of 1.26 Å, while the distance between the C–C bonds in the terminal CH_3_ group is 1.46 Å [68]. Thus, in the case of DPPC, which consists of two chains of palmitic acid of 16 carbon atoms, the length occupied by them will be 40.4 Å. This is the length corresponding to the stretched hydrocarbon chains (without tilt) of a DPPC molecule. It is known, however, that in the gel phase of DPPC bilayers, the hydrocarbon chains are tilted at an angle, θ_tilt_, of approximately 32° [69] relative to the plane of the membrane, to fill the created free space by polar groups and to increase the proximity between adjacent chains, maximizing van der Waals interactions [69]. Thus, given that the chains are tilted, the thickness of the bilayer will not be 40.4 Å, but rather 40.4 × cos(32°) = 34.3 Å, and the difference between the thickness corresponding to the stretched and tilted chains is given by 40.4 − 34.3 = 6.1 Å. Therefore, the average increase in d values obtained at the ripple and gel phase of DPPC upon addition of DCF is similar (within the error) to what it is expected for chains’ untilting. This conclusion can be further confirmed by comparison of WAXS patterns obtained for DPPC and DPPC upon DCF addition (Figure 7A). The chain packing of DPPC bilayers is significantly influenced by DCF by the appearance of one Bragg peak (Figure 7A, upper diffraction WAXS pattern) instead of the typical two Bragg peaks (Figure 7A, lower diffraction WAXS pattern). This indicates a change from the orthorhombic unit cell of tilted chains to a hexagonal packing of non-tilted chains, which correlates well with the already-discussed increase in d values observed in SAXS.

Figure 7B illustrates DCF interaction with DPPC at the gel phase possibly inducing a change of the polar headgroups’ orientation in a perpendicular position to the bilayer plane, reducing the area requirement of the headgroups and allowing the chains to be oriented in an upright position. This schematic location of DCF in the headgroup region is further confirmed by the calculation of the cross-sectional area (*A*_0_) of a single aliphatic chain obtained from WAXS patterns in the gel phase (Supplementary Table S2). *A*_0_ of 20.1 Å^2^/aliphatic chain was determined for the DPPC, which is in good agreement with the previously reported [23]. Addition of DCF to DPPC reduces *A*_0_ to 19.0 Å^2^/aliphatic chain, ruling out the possibility of penetration of DCF within the aliphatic core of the membrane as previously observed for other drugs [70].

## 3. Discussion

Despite being widely prescribed, DCF has been associated with cases of severe GI injury, especially when administrated on a daily basis for the treatment of chronic situations [4].

To investigate the effects of DCF on the GI phospholipid lining, we have methodically examined the interaction between DCF and PC membranes at relevant lipid membrane phases using a selection of biophysical techniques that offer complementary information. These techniques clearly proved, at a pH of 5.0, that DCF presents a noticeable distribution at the membrane phase despite being partially ionized. Furthermore, we have concluded that the chemical association between DCF and the membrane phospholipids is clearly pH dependent, highlighting the importance of the pH of studies in understanding NSAID–PC interactions. This pH dependence is consistent with the findings reported in studies involving other NSAIDs (e.g., aspirin, ibuprofen, naproxen, tolmetin, indomethacin, nimesulide, acemetacin, piroxicam, meloxicam, and celecoxib) [7,10,27,28,29,71].

Besides quantifying DCF membrane distribution, we have also been able to propose a molecular orientation of this NSAID within the lipid bilayer. DCF is located at the phospholipid headgroup region, and probably anchored at the choline level by an electrostatic interaction with the negatively charged carboxylate moiety. DCF preferential location at the membrane polar region was also observed by Ferreira et al., who determined the distribution of the drug in egg lecithin membrane models by spectroscopic techniques while calculating surface concentrations of the drug by zeta-potential measurements [45]. In another study, Manrique-Moreno et al. presented an interesting contribution to understanding the membrane-activity of some NSAIDs such as DCF, ibuprofen, and naproxen, using different spectroscopic and thermodynamic techniques. Regarding DCF, this study proposed a location in closer proximity to the phosphate region [25]. The observations described in these studies are thus consistent with our DCF proposed location at the L_α_ phase. Additionally, our quenching studies also support the hypothesis that DCF can penetrate the GI protective phospholipid gel lining as the drug was able to reach the membrane-located fluorophores even at the more ordered L_β’_ phases. Interestingly, the quenching efficiency of the membrane fluorophore was significantly higher in the gel phase, indicating that this more ordered phase does not hinder DCF membrane penetration, which is consistent with the proposed topical injury to the GI mucosa.

Direct techniques (DLS and DSC) that do not require the insertion of fluorophores in the membrane confirmed DCF location at the headgroup lipid region and suggested a membrane fluidizing effect of the drug. SAXS and WAXS studies confirmed DCF’s strong effect on membrane structure observed by loss of correlation between the lipid bilayers and membrane-fluidizing effect promoted by the drug. Additionally, the measured lipid spacings in the gel phase indicated that DCF penetrates at the lipid polar region, allowing the phospholipid acyl chains to lose their tilt. In vivo, these changes in lipid packing can open spaces for the passage of gastric acid and other aggressive factors that may injure the GI mucosa (as schematically represented in Figure 1B).

In susceptible patients where the phospholipid protective lining may have lost part of its gel integrity arrangement and may present fluid domains, DCF is also able to induce structural changes. At this fluid domains DCF increases the hydration layer, which is consistent with its capability to attenuate the hydrophobic barrier properties of the upper GI tract. Such a biophysical effect of increasing the GI phospholipid lining wettability has been described as able to promote the back diffusion of luminal acid into the mucosa and the development of erosions [72].

In agreement with the current work, some studies confirmed the disordering and/or fluidizing effect of DCF in lipid membranes and linked the results to other toxic events. Suwalsky et al. assessed the influence of DCF in a class of lipids found in the outer moiety of the erythrocyte membranes and in isolated human erythrocytes membranes using X-ray diffraction, fluorescence spectroscopy, and scanning electron microscopy [45]. The authors concluded that DCF interacted with red cell membranes, inducing a membrane disordering effect and changes in normal cellular morphology, which could presumably contribute to DCF toxicity effects [45]. A membrane disordering effect induced by DCF was also observed by Souza et al. [44] in a study where liposomes and monolayers made of soy bean phosphatidylcholine where used to evaluate drug–lipid interaction [44]. The authors concluded that this membrane disordering effect could be related to myotoxicity. Manrique-Moreno et al. also proposed that DCF induced a membrane fluidizing effect and hydration changes of the lipid polar headgroups which could in turn have consequences at the membrane level, such as changes in semipermeable properties, cell growth, and modulation of the activity of membrane associated-enzymes [25].

DCF promotes calcein release in liposomes prepared with the main classes of lipids present in the IMM, suggesting a membrane permeabilization either by a decrease in membrane microviscosity or by induction of lateral phase separation with the advent of transient spaces at the interfacial regions that allow calcein leaking. The capacity of DCF to induce this effect must be related to the drug location at the headgroup region of IMM membrane models perturbing the lipid packing of this ordered membrane region. The high value of *θ* measured in IMM L_α_ phases before addition of DCF confirms the high level of lipid order in the polar regions of this membrane model, probably due to CL anionic groups interacting with Ca^2+^ divalent cations. It is at this level that DCF disordering effects are more evident, also possibly owing to its negatively charged carboxylate group impairing Ca^2+^ coordination, which may create a repulsive effect between the phospholipid headgroups. The membrane permeabilization induced by DCF observed in our study can be further related to the MPT effect at the mitochondrial membrane reported for this drug [18,19,32,33]. As observed for calcein release studies, the increase in mitochondrial permeability may result from pore formation through which ions can diffuse. The dissipation of the proton gradient across a permeable IMM can then result in membrane depolarization and in impairment of ATP synthesis [18,19,32,33]. Ultimately, these effects will lead to damaging of GI intercellular junctions and GI toxicity (as schematically represented in Figure 1B) [6,11].

Furthermore, DCF preferential location at the headgroup region of the H_II_ phases of the IMM model is a synonym of a drug appetence for the contact points of mitochondria membranes. At these sites, the insertion of DCF carboxylate group may also create a repulsive effect, inducing a positive lateral pressure and disordering effect (also confirmed by the decrease of *θ* values with DCF concentration). By inducing a positive lateral pressure, DCF can work as a bilayer stabilizer [73] reducing the high negative curvature regions where the contact points between IMM and OMM occur. This interference with membrane dynamics at the contact points of mitochondrial membranes has been described for other compounds that act as bilayer stabilizers or H_II_ phase promoters, and has been pointed out as a cause of impairing H^+^-ATPase activity [74]. Moreover, the disturbance of membrane lipid dynamics at mitochondria membrane contact points can be also related to DCF’s mitochondrial toxicity by MPT-associated processes. This mitochondrial toxicity is very likely the pathway involved in DCF-induced apoptosis, and the cumulative damage to mitochondrial function seems a plausible putative mechanism to explain DCF hepatic, renal and cardiovascular deleterious effects that cannot be explained by the COX inhibition mechanism [6,21,22,23,24,25,26,27,28,29,30].

Other studies reported the drug-interaction effects on membrane lipid organization, using models mimicking the mitochondrial membrane and/or the native mitochondrial membrane, with different drugs, namely piroxicam, nimesulide, menadione, and *p*-trifluoromethoxyphenylhydrazone (FCCP) [57,58,59,60]. Generally, all the tested drugs interacted with the lipid membrane systems, altering their structure and dynamics with several consequences: increase of membrane permeability, a lipid disordering effect, changes in lipid phase transitions, and membrane fusion effects [57,58,59,60]. These observed biophysical effects also suggested a change in the curvature and elastic properties of the membrane models and were coherent with the drugs’ mitochondrial toxic effects [57,58,59,60].

In summary, the DCF interactions with GI phospholipid model systems and IMM models demonstrated in this work provide a novel molecular and biophysical perspective to explain the reported topical harmful actions of DCF that could not be solely explained as resultant from COX inhibition. Although several interesting and important studies have used animal and cellular models to understand GI and mitochondrial toxicity of DCF [2,5,18,19,36,37,48,56,75], the use of lipid models avoids the complexity of biological systems while providing a basis for evaluating the structural and biophysical changes on lipid bilayers that are relevant for the functionality of native membranes. Our work was carefully planned regarding the lipid systems used, as well as the conditions of study, to provide a pertinent model for the lipid environment encountered by the drug in the biological milieu. The concentrations of DCF studied (40 µM and the range 0–80 µM) were also chosen to be relevant in a context of chronic use and toxicity level, while not excessively surpassing the drug plasmatic levels (1.8–24.2 µM); this took into consideration the fact that NSAIDs reach particularly high concentrations in tissues in which they cause effects and side effects and that we cannot take systemic concentrations as surrogates for GI and mitochondrial concentrations [18,76]. Additionally, sub-cytotoxic concentrations of DCF (<100 µM) were described as able to cause mitochondrial dysfunction [48], and thus we used this as the upper reference for the concentration range studied.

Future research directions, to complement the current work, could involve the study of DCF interactions with lipid membranes by molecular dynamics (MD) simulations. Indeed, adsorption of small molecules on lipid membranes has been studied by MD simulations to predict the effect of the adsorbed agent in lipid membrane curvature [77]. Therefore, MD simulations could be applied to study the effects of DCF in local membrane curvature, as this would be helpful to corroborate its effect at the contact points of mitochondrial membranes. Furthermore, MD simulations might be very useful for probing the molecular mechanism of membrane permeation processes [78] which is also relevant to understand DCF interaction with GI lipid lining. Besides MD simulation studies, other experimental approaches that could add relevant information to this work are time-resolved fluorescence anisotropy for studying the restricted rotational diffusion of probes in membrane model systems. These studies would allow evaluating DCF’s effect on the membrane microviscosity at L_β’_ phases as the surrounding ordered media usually imposes certain restrictions on the orientations of the probe. Assays involving the SAXS and WAXS studies of DCF and other NSAIDS in IMM models are also very relevant and currently underway, and future assays with mitochondrial extracted membranes are also planned.

At this point, the study herein presented provides new elements for understanding previous literature results regarding DCF toxicity and, from a molecular point of view, the additional knowledge gained about DCF–membrane interaction may aid in the future design of safer NSAIDs for chronic therapeutic purposes. Strategies to minimize a unique biochemical action of NSAIDs, such as by coadministration of a phospholipid, esterification of NSAIDs (with or without the addition of hydrogen sulfite or nitric oxide moieties), or use of selective COX2 inhibitors, have proven to be ineffective in eliminating their toxicity. Strategies that affect NSAIDs’ topical actions, could be a more realistic approach for reducing toxicity, namely by developing acid resistant nanoformulations that carry the drug, protecting it from interacting with the lipid membranes at GI tract, and avoiding biophysical impairment of mitochondrial membranes.

## 4. Materials and Methods

### 4.1. Materials

The lipids—DMPC, DPPC, DOPC, DOPE, and CL—were obtained from Avanti Polar Lipids Inc. (INstruchemie, Delfzyl, The Netherlands) at a purity grade of >99% and were used without further purification. Nonsteroidal anti-inflammatory drug, DCF, and the probes DPH and calcein were purchased from Sigma-Aldrich Química, S.L. (Sintra, Portugal). All other chemicals were purchased either from Sigma-Aldrich Química, S.L. or Cymit Quimica (Barcelona, Spain) and were of the highest commercially available purity. Drug solutions were prepared either with Hepes buffer (pH 7.4) or acetate buffer (pH 5.0).

### 4.2. Preparation and Labeling of Lipid Model Systems

Preparation of the lipid model systems was made by the lipid film hydration method followed by extrusion previously reported [58,59,60]. Briefly, adequate portions of DMPC, DPPC, or ternary mixtures of DOPC:DOPE:CL in a 1:1:1 molar ratio were dissolved in chloroform, and subsequently evaporated to dryness in a rotary evaporator. The dry lipid films were hydrated with an adequate volume of buffer (pH 5.0 for DPPC and DMPC, and pH 7.4 in the case of the ternary mixture to which a small volume of CaCl_2_ to reach a 1:1 molar ratio of Ca^2+^:CL was also added) to achieve a final lipid concentration up to 1500 μM, and vortexed 20 min. The lipid film hydration was performed at a temperature above the *T*_m_ of the lipid or of the lipid mixture (37 °C for DMPC and ternary mixture and 55 °C for DPPC). Lipid suspensions were then submitted to several freeze–thaw cycles and extruded through 100 nm nucleopore polycarbonate filters (Millipore SAS, Molsheim, France) to produce LUVs.

To obtain labeled lipid model systems, methanolic stock solutions of fluorescent probes were added to the chloroform solutions of lipids in an amount such that the lipid:probe molar ratio is always greater than 300:1, to prevent structural changes of the membrane model system. After addition of the probe, lipid model systems were prepared by the lipid film hydration method followed by extrusion described above. Increasing concentrations of DCF were added to LUVs (final concentration of 1500 μM) labeled with TMA-DPH or DPH probes to obtain final drug concentration of 0–80 μM. After mild shaking, the labeled LUVs suspensions were protected from light and incubated for 2 h prior to measurements.

For calcein leakage studies, membrane model systems were prepared by the same lipid film hydration method followed by extrusion where calcein (30 mM) was added in the buffer during the hydration step. After the extrusion process, a molecular exclusion chromatography was performed to exclude the excess of free probe from LUVs. Increasing concentrations of DCF were added to LUV suspensions (final concentration of 1500 μM) containing calcein to obtain final drug concentration of 0–80 μM.

### 4.3. Derivative Spectrophotometry Studies

K_d_ of DCF was determined in LUVs of DMPC at pH 5.0. A series of samples containing increasing concentrations of LUVs (0–1 mM) and a fixed concentration of DCF (40 µM) was prepared. A set of reference samples was prepared identically but without the drug. The samples and references were vortexed, mixed, and incubated at 37 °C.

The absorption spectra of samples and references were plotted in the 250–500 nm range, on a Lambda 45 UV-Vis spectrophotometer (Perkin-Elmer, Villepinte, France) equipped with a thermostated cell holder at 37.0 ± 0.1 °C. Derivative spectra were made from absorption spectra after subtracting the absorption of the references and using an Excel^®^-based routine previously published [79]. Third-derivative spectra were determined to eliminate the spectral interferences due to light scattered by the lipid suspensions and to improve the resolution of spectral bands. By representing the λ_max_ or λ_min_ values of the third-derivative spectra as a function of the concentration of LUVs ([*DMPC*]), a non-linear regression was obtained from which it was possible to determine *K*_d_:

(1) $$\frac{d^{3}{\mathrm{Abs}}_{T}}{{d\lambda }^{3}}\;=\;\frac{d^{3}{\mathrm{Abs}}_{a}\;}{{d\lambda }^{3}}+\;\frac{\left(\frac{d^{3}{\mathrm{Abs}}_{l}}{{d\lambda }^{3}}\;-\;\frac{d^{3}{\mathrm{Abs}}_{a}}{{d\lambda }^{3}}\right)K_{d}{[\mathrm{DMPC}]V}_{l}}{{1\;+\;K}_{d}{[\mathrm{DMPC}]V}_{l}}$$

In the fitting expression, *Abs*_T_, *Abs*_a_, and *Abs*_l_ stand for total absorbance of the drug (in lipid and aqueous media), absorbance of the drug in the aqueous media, and absorbance of the drug in the lipid media, respectively. The LUV concentration, [*DMPC*] expressed in mol·L^−1^, is multiplied by the lipid molar volume in L·mol^−1^ (*V*_l_) to obtain a dimensionless value of *K*_d_ that can be compared with others values described in the literature for other systems [50].

### 4.4. Fluorescence Quenching Studies

The fluorescence excitation and emission spectra of LUVs of DMPC or DOPC:DOPE:CL (1:1:1) labeled with TMA-DPH and DPH probes were acquired using a Perkin-Elmer LS-50B spectrofluorimeter using quartz cells of 10 mm optical path thermostated at 10.0, 30.0, 37.0, and 45.0 ± 0.1 °C (for DMPC) or 25.0 and 70.0 ± 0.1 °C for ternary lipid mixture. In the case of DMPC systems, temperatures were selected to evaluate the type of quenching mechanism established between the probe and the drug, and to evaluate the drug location at two lipid phases: L_β’_ or gel phase present at temperatures below *T*_m_ of DMPC, i.e., <23 °C, and L_α_ or fluid phase for the temperatures above *T*_m_ of DMPC, i.e., >23 °C. These phases were studied for representing the disordered and ordered regions that might be found in the GI phospholipid lining. In the case of the ternary lipid model, temperatures were chosen to evaluate drug location at the lipid lamellar and non-lamellar phases that can be found in IMM: L_α_ (at 25.0 °C) and H_II_ (at 70.0 °C) [58,59,60].

The excitation wavelength was defined to 360 nm and emission wavelength was set to 427 nm with excitation and emission slits of 5 nm and a scan rate of 400 nm min^−1^. Fluorescence values were corrected for light scattering contributions by subtracting the intensities from unlabeled samples at the same conditions; such contributions were always negligible (less than 0.5%). All fluorescence intensity data were corrected from absorption and inner filter effects [55]. Fluorescence lifetime measurements were made with a Fluorolog Tau-3 Lifetime system (HORIBA Scientific, Piscataway, NJ, USA). Modulation frequencies were acquired between 2 and 110 MHz. Integration time was 8 s. Manual slits used were 0.5 mm, slits for excitation and emission monochromator were 7.000 All measurements were made using Ludox as a reference standard (τ = 0.00 ns).

The extent of fluorescence quenching induced by the quencher (DCF) in both probes (TMA-DPH and DPH) was evaluated by Stern–Volmer constants (*K*_sv_) obtained by the Stern–Volmer linear plots:

(2) $$\frac{I_{0}}{I}\;\mathrm{or}\;\frac{\tau_{0}}{\tau }{=\;1\;+\;K}_{\mathrm{sv}}{\left[\mathrm{DCF}\right]}_{m}$$

where (*I*, *τ*) and (*I*_0_, *τ*_0_) are, respectively, the steady-state fluorescence emission and lifetime of the probe in the presence or in the absence of the drug. Since only drug molecules distributed in the membrane can quench the probes inserted in LUVs’ bilayers, membrane concentrations of the drug within the membrane ([*DCF*]_m_) were used instead of total concentration of the drug added to the labeled systems (0–80 µM). DCF membrane concentrations were calculated from total drug concentrations added using the following equation [55,60]:

(3) $${\left[\mathrm{DCF}\right]}_{m}\;=\;\frac{K_{d}{\left[\mathrm{DCF}\right]}_{T}}{K_{d}V_{m}+\;(1\;-V_{m})}$$

where *K*_d_ is the distribution coefficient of DCF in the lipid system (calculated as described in Section 4.1) and *V*_m_ is the membrane volume fraction [55,60].

The efficacy of DCF to quench the fluorescence of each probe was evaluated by the bimolecular constant, *K*_q_ [50,55]:

(4) $$K_{q}\;=\;\frac{K_{\mathrm{sv}}}{\tau_{0}}$$

*K*_q_ is independent of intrinsic microenvironment changes sensed by each probe, which are reflected in the different values of *τ*_0_ (i.e., lifetime of the excited state characteristic of each probe [50,55]) and was used to infer the relative location of DCF (quencher) in the lipid model system.

### 4.5. Fluorescence Anisotropy Studies

Steady-state anisotropy of TMA-DPH and DPH probes included in DOPC:DOPE:CL (1:1:1) systems in the presence of DCF (0–80 µM) was measured in a LS-50B spectrofluorimeter (Perkin-Elmer, Villepinte, France), with quartz cell with an optical path of 10 mm, thermostated at 25.0 ± 0.1 °C or 70.0 ± 0.1 °C (for mimicking, respectively, L_α_ and H_II_ phases of IMM) with automatic insertion of vertical and horizontal polarizers. The probes were excited with vertical polarized light at a wavelength of 360 nm and resultant fluorescence intensities were recorded at a wavelength of 427 nm, with the emission polarizer oriented parallel (*I*_∥_) and perpendicular (*I*_⊥_) to the excitation polarizer allowing the determination of steady-state anisotropy (*r*_ss_):

(5) $$r_{\mathrm{ss}\;}\;=\;\frac{I_{∥}\;-{\;\mathrm{GI}}_{⊥}}{I_{∥}\;+\;2{\mathrm{GI}}_{⊥}}$$

The grating factor *G* is an instrumental preference of the emission optics for the horizontal orientation to the vertical orientation. It can be measured by moving the excitation polarizer to the horizontal orientation and comparing the intensities when the emission polarizer is vertically and horizontally polarized, respectively.

The rotational correlation time (*θ*) of the probes inserted in the ternary lipid model system was calculated from the steady-state fluorescence anisotropy, $r_{\mathrm{ss}}$, values using the following equation [50,55,59]:

(6) $$\theta \;=\;\tau \left(\frac{r_{\mathrm{ss}}\;-{\;r}_{\infty }}{r_{0}-r_{\mathrm{ss}}}\right)$$

where *τ* are the measured lifetime values of each probe during the excited state and *r*_0_ is the fundamental anisotropy (considered to be 0.362) [80]. We can assume the limiting anisotropy as *r*_∞_ ≈ 0 since the rotational motion of the probe is not significantly hindered at the membrane fluid phase (condition assured by making the measurements at temperatures above the *T*_m_ of the lipid system). *θ* gives indications about probe motion, and indirectly reports the microviscosity of the lipid environment surrounding the probe and how is it affected by the increasing concentrations of the DCF (0–80 µM).

### 4.6. Calcein Leakage Studies

LUVs of DOPC:DOPE:CL (1:1:1) entrapping calcein were prepared as described in Section 4.1 and were incubated with DCF (0–80 µM) for 10 min at 25 °C. Membrane destabilization accompanied by calcein release was monitored in a LS-50B spectrofluorimeter (Perkin-Elmer, Villepinte, France) by an increase of the fluorescence emission intensity at 520 nm upon excitation at 490 nm. Total (100%) amount of calcein was determined after disrupting the lipid model systems upon addition of Triton X-100. The results of the leakage experiments are presented as the calcein release (%), calculated according to [58,60]:

(7) $$\mathrm{Calcein}\;\mathrm{release}\;(\%)\;=\;\frac{100(I_{x}-I_{c})}{I_{T}}$$

where *I*_x_ is the fluorescence intensity with x DCF concentration, *I*_c_ is the control fluorescence (i.e., fluorescence of the lipid system in the absence of DCF), and *I*_T_ is the maximum fluorescence value reached upon lysis of the membrane systems.

### 4.7. Differential Scanning Calorimetry Studies

DSC measurements were performed using a microcalorimetry system (MicroCal VP-DSC, Malvern Panalytical, Paralab, Lisbon, Portugal) as described elsewhere [23,81]. LUVs of DMPC (≈1 mg mL^−1^ resulting in a final lipid concentration of 1500 µM) with or without DCF (40 µM) and the buffer (pH 5.0) used as reference were degassed before measurement and then were transferred to the DSC sample holder. During the measurements, the samples were kept under nitrogen overpressure. The scan rate employed was 1 °C min^−1^ in the temperature range between 15 and 35 °C after an initial isothermal period of 15 min. The obtained data were analyzed using MicroCal Origin software. After the calorimetric scans, aliquots of the scanned samples were used to determine the amount of phospholipid by phosphorus assay [82].

### 4.8. Dynamic Light Scattering Studies

The main phase transition temperature and the cooperativity of LUVs of DMPC (1500 µM) with or without DCF (40 µM) at pH 5.0 was determined by DLS, as already described in previously reported studies [29,83]. The experiments were performed in a Zetasizer Nano ZS (Malvern Panalytical, Paralab, Lisbon, Portugal) containing a controlled temperature cell holder. The samples were heated from 10 to 40 °C with intervals of 1 °C with an equilibration period of 2 min. At each temperature, 3 runs of 2 min were made. The results were collected as average count rate versus temperature (*T* in °C) and the data were fitted using the following equation [29,83]:

(8) $$\mathrm{Count}\;\mathrm{rate}\;=\;b_{1}+m_{1}T\;+\frac{b_{2}-b_{1}+m_{2}T-m_{1}T}{1+10^{B(1/T-1/T_{m})}}$$

where *m*_1_ and *m*_2_ are the slopes of the linear fits to the data before and after the phase transition region, respectively, and *p*_1_ and *p*_2_ are the corresponding y intercepts. From the fitted equation, *T*_m_ and cooperativity (B) of the phase transition were obtained.

### 4.9. Syncrotron Small- and Wide-Angle X-ray Scattering Studies

For the X-ray scattering experiments, samples consisted of lipid dispersions of DPPC which had not been extruded and was prepared as described in previous works [23,26,29,83]. Dispersions of DPPC prepared in buffer of pH 5.0 in the absence or presence of DCF (40 µM) were transferred into X-ray transparent glass capillaries with 1.5 mm diameter (Hilgenberg, Malsfeld, Germany), which were sealed using a flame and stored at 4 °C until measurement. SAXS and WAXS scattering measurements were recorded at the Austrian SAXS/WAXS beamline at the synchrotron light source ELETTRA (Trieste, Italy) employing a monochromatic synchrotron radiation with wavelength of 1.54 Å and X-ray energy of 8 keV. WAXS and SAXS patterns were recorded using a Pilatus 100K 2D and Pilatus 1M detector system, respectively, with a pixel size of 172 µm at positions that covered the typical diffraction spacing range (*s* = 2 sin θ/λ, where λ is the wavelength and 2θ is the scattering angle) of interest. The diffraction spacings were calibrated using the lamellar peaks of silver behenate (SAXS) and *p*-bromo benzoic acid (WAXS) as standards. Measurements were performed at temperatures between 20 and 50 °C thermostated with a water bath (stability ±0.1 °C, Unistat CC, Huber, Offenburg, Germany) to obtain the diffraction patterns typical of DPPC lipid phases (L_β’_, P_β’_, and L_α_) and evaluate the effect of DCF in such phases. After each temperature step, the sample was equilibrated for 5 min before the SAXS and WAXS diffraction patterns were recorded for 30 s every minute. A shutter mounted before the sample was kept closed when no data were acquired to minimize the X-ray exposure to the sample. The integration of the scattered intensity and the primary data reduction was performed using the program Fit2D. After the raw data had been corrected for detector efficiency, and the background scattering both from water and the sample cell had been subtracted, all Bragg peaks were fitted by Lorentzian distributions using Microcal Origin software. In each respective phase regime, only the strongest reflections were considered. Each final diffraction pattern is presented as normalized scattering intensity in arbitrary units versus the reciprocal spacing (*s*). *fwhm* were determined and used to calculate the correlation length between the lipid bilayers (*ξ* = 2π/*fwhm*). The repeat distances, *d* (*d* = 1/*s*) were calculated from the peak positions of the diffraction patterns and given as an average value of all the repeat distances measured.

From the WAXS patterns, it was possible to calculate the cross-sectional area of a single aliphatic chain (*A*_0_). In the L_β’_ phase of the pure DPPC system, the unit cell is known to be orthorhombic and this unit cell contains two aliphatic chains. The two parameters *a* and *b* of the orthorhombic cell are calculated using the following equations:

(9) $${a\;=\;2d}_{20}$$

(10) $$b\;=\;d_{11}/\sqrt{\left(1-{\left(d_{11}/2d_{20}\right)}^{2}\right)}$$

in which *d*_11_ and *d*_20_ are the two distances determined by the peak positions on the X-ray pattern. The sharp peak is attributed to *d*_20_ and the large peak to *d*_11_. In the samples where a symmetric peak indicates that the chains are packed in a hexagonal lattice, the unit cell parameter *a* can be calculated by:

(11) $$a=\;d_{20}2/\sqrt{3}$$

In this case of a hexagonal unit cell, the surface of the cell (*a* × *d*_10_) is directly the cross-section area of one aliphatic chain.

## Figures and Tables

**Table 1:** Distribution coefficients of DCF obtained in a biphasic membrane/aqueous system.

pH	Δλ	*K*_d_	Log *K*_d_
3.0		26,000 ± 3000 ^1^	4.41 ± 0.05 ^1,^***
5.0	319–328	6970 ± 379 ^2^	3.84 ± 0.02 ^2,^***
7.4		1200 ± 100 ^1^	3.08 ± 0.04 ^1,^***

^1^ Values reported in [43] calculated by derivative spectrophotometry in LUVs of egg-phosphatidyl choline (EPC). ^2^ Values determined by derivative spectrophotometry in LUVs of DMPC. Results are the mean ± standard deviation of at least three independent measurements and comparisons were performed using one-way ANOVA with the Tukey-Kramer post-test; *** indicates a value of *p* < 0.001, which was considered statistically significant.

**Table 2:** Bimolecular quenching rate constant (*K*_q_) obtained from measurements of fluorescence quenching of TMA-DPH and DPH by DCF in two membrane model systems at pH 5.0 (in the case of GI mimicking) and pH 7.4 (in the case of IMM mimicking).

**GI mimicking: PC Membrane Model**		
**Probe**	**Lipid Phase**	***K*_q_ × 10^9^ (M^−1^·s^−1^)**
**TMA-DPH**	L_β′_	2.63 ± 0.03
	L_α_	2.30 ± 0.09 ***
**DPH**	L_β′_	0.37 ± 0.02 ***
	L_α_	1.34 ± 0.04 ***
**IMM mimicking: DOPC:DOPE:CL (1:1:1) Membrane Model**		
**Probe**	**Lipid Phase**	***K*_q_ × 10^9^ (M^−1^·s^−1^)**
**TMA-DPH**	L_α_	1.24 ± 0.06 ***
	H_II_	2.90 ± 0.23 ***
**DPH**	L_α_	0.73 ± 0.04 *
	H_II_	1.87 ± 0.29 ***

L_α_, L_β′_ and H_II_ stand, respectively, for lamellar fluid phase, lamellar gel phase, and inverted hexagonal phase. Results are the mean ± standard deviation of at least three independent measurements and comparisons were performed using one-way ANOVA with the Tukey-Kramer post-test for the following paired observations: (1) fluid phase of TMA-DPH or DPH vs. gel phase of TMA-DPH or DPH in GI mimicking (***, *p* < 0.001); (2) gel phase of DPH vs. gel phase of TMA-DPH in GI mimicking (***, *p* < 0.001); (3) fluid phase of DPH vs. fluid phase of TMA-DPH in GI mimicking (***, *p* < 0.001); (4) inverted hexagonal phase of TMA-DPH vs. fluid phase of TMA-DPH in IMM mimicking (***, *p* < 0.001); (5) inverted hexagonal phase of DPH vs. fluid phase of DPH in IMM mimicking (***, *p* < 0.001); (6) fluid phase of DPH vs. fluid phase of TMA-DPH in IMM mimicking (*, *p* < 0.05); (7) inverted hexagonal phase of DPH vs. inverted hexagonal phase of TMA-DPH in IMM mimicking (***, *p* < 0.001); (7) fluid phase of TMA-DPH in IMM mimicking vs. fluid phase of TMA-DPH in GI mimicking (***, *p* < 0.001); and (8) fluid phase of DPH in IMM mimicking vs. fluid phase of DPH in GI mimicking (***, *p* < 0.00)1. DOPC, DOPE, and CL are 1,2-dioleoyl-*sn*-glycero-3-phosphocholine and 1,2-dioleoyl-*sn*-glycero-3-phosphoethanolamine, and Cardiolipin, respectively.

## Supplementary Materials

Supplementary materials can be found at http://www.mdpi.com/1422-0067/19/11/3411/s1.

## Abbreviations

*A*_0_	Cross-sectional area of a single aliphatic chain
*Abs*_a_	Absorbance of the drug in the aqueous media
*Abs*_T_	Total absorbance of the drug
*Abs*_l_	Absorbance of the drug in the lipid media
ATP	Adenosine triphosphate
*B*	Cooperativity of the main phase transition
CL	Cardiolipin
*ξ*	Correlation length
COX	Cyclooxygenase
*d*	Long or short spacing
DCF	Diclofenac or sodium 2-[2-(2,6-dichloroaniline)phenyl]acetate
DLS	Dynamic Light Scattering
DMPC	1,2-dimyristoyl-*sn*-glycero-3-phosphocholine
DOPC	1,2-dioleoyl-*sn*-glycero-3-phosphocholine
DOPE	1,2-dioleoyl-*sn*-glycero-3-phosphoethanolamine
DPH	1,6-diphenyl-1,3,5-hexatriene
DPPC	1,2-dipalmitoyl-*sn*-glycero-3-phosphocholine
DSC	Differential Scanning Calorimetry
EPC	Egg phosphatidylcholine
*fwhm*	full width of the peaks at one-half of their intensity
GI	Gastrointestinal
H_II_	Inverted hexagonal phase
*I*_c_	Control fluorescence
IMM	Inner mitochondrial membrane
*I*_T_	Maximum fluorescence value
*I*_x_	Fluorescence intensity at a given drug concentration
*K*_d_	Distribution coefficient
*K*_q_	Bimolecular quenching rate constant
*K*_SV_	Stern–Volmer constant
L_α_	Lamellar fluid phase
L_β’_	Lamellar gel phase (tilted chains)
L_β_	Lamellar gel phase (untilted chains)
LUVs	Large Unilamelar Vesicles
MPT	Mitochondrial permeability transition pore
NSAID	Non-steroidal anti-inflammatory drug
OMM	Outer mitochondrial membrane
P_β’_	Ripple phase
PC	Phosphatidylcholine
SAXS	Small angle X-ray scattering
*θ*	Rotational correlation time
*r*_ss_	Steady-state fluorescence anisotropy
*r*_0_	Fundamental anisotropy
*τ*_0_	Lifetime of the excited state
*T*_m_	Main phase transition temperature
TMA-DPH	*N,N,N*-Trimethyl-4-(6-phenyl-1,3,5-hexatrien-1-yl)phenylammonium *p*-toluenesulfonate
*T*_p_	Pre-transition temperature
TRP	Transient receptor potential
*V*_m_	Membrane volume fraction
*V*_l_	Lipid molar volume
WAXS	Wide Angle X-ray Scattering
