# Supplementary material for: A Molecular Biophysical Approach to Diclofenac Topical Gastrointestinal Damage
Source: Int J Mol Sci. 2018 Oct 31;19(11):3411. doi: 10.3390/ijms19113411 (PMC6275047; doi:10.3390/ijms19113411)
Supplement: Supplementary file 1 [file ijms-19-03411-s001.pdf]

Supplementary materials

A molecular biophysical approach to diclofenac topical gastrointestinal damage

Diclofenac location in a membrane/aqueous system

Fluorescence quenching data obtained by intensity measurements alone can be explained by either dynamic (collisional) or static processes. In the case of collisional quenching, molecular collisions occur between the quencher and fluorophore for which the quencher must diffuse to the fluorophore during the lifetime of the excited state. Upon contact, the fluorophore returns to the ground state, without emission of a photon. In static quenching a complex is formed between the fluorophore and the quencher, and this complex is nonfluorescent. To evaluate the type of quenching mechanism established between the quencher drug DCF and the fluorophore incorporated in the membrane model system (TMA-DPH or DPH probes) steady-state fluorescence measurements were made at several temperatures and Stern-Volmer constants (K_{SV}) were calculated using Equation 3 (Table S1). For DPH, K_{SV} values increased with increasing temperatures indicating a collisional quenching mechanism. Indeed, collisional quenching occurs from the molecular encounters between fluorophore and quencher. Hence, higher temperatures result in faster diffusion and thus larger amounts of collisional quenching. For TMA-DPH, K_{SV} values decreased with increasing temperatures indicating a static quenching mechanism. This occurs because higher temperature will typically result in the dissociation of weakly bound complexes, and hence smaller amounts of static quenching. This was further confirmed by lifetime measurements that, in the case of TMA-DPH, have shown no variation of τ values of the probe (τ_0/τ=1) as illustrated in Figure 3 – C.

Table S1. Stern-Volmer constant (K_{SV}) obtained from measurements of fluorescence quenching of TMA-DPH and DPH by DCF in GI mimetic model systems at pH 5.0.

Probe	Temperature (°C)	K_{sv} (M^{-1})	Quenching mechanism
TMA-DPH	10	13.92 ± 0.15	Temp↑ ↓K_{sv}<br>τ_0/τ=1<br>Static quenching
	30	10.86 ± 0.35	
	37	9.96 ± 0.30	
	45	8.61 ± 0.44	
DPH	10	3.28 ± 0.20	Temp↑ ↑K_{sv}<br>$\tau_0/\tau=I_0/I$<br>Collisional quenching
	30	8.35 ± 0.31	
	37	11.69 ± 0.37	
	45	15.89 ± 0.56	

Effects of Diclofenac in the microviscosity of the membrane

The DSC analysis was performed to evaluate the interactions of DCF with a PC rich mimetic system of the GI lining protective layer. Therefore, the pH of analysis was pH 5.0 to simulate the acidic pH of GI tract where NSAIDs absorption occurs, and the gastric pH in a fed state according to NSAIDs posology regime. The thermograms obtained show the heat flow associated with the lipid phase transitions, and it is possible to evaluate the effect of the drug altering the thermotropic behavior of lipid membrane. The thermogram obtained for pure DMPC system shows two endothermic peaks. The smaller endothermic peak occurs at lower temperatures (15.4 °C) and corresponds to the pretransition (T_p) between the gel phase ($L_{\beta'}$) and the ripple phase ($P_{\beta'}$). The larger endothermic peak occurring at higher temperatures (24.50 °C) corresponds to the main transition (T_m) between the ripple phase ($P_{\beta'}$) and the fluid phase (L_α). When DCF was added to the system, the gel to fluid phase transition still occurred but was broadened and slightly shifted to lower temperatures (24.16 °C), whereas the pretransition disappeared. The cooperativity of the main phase transition was also reduced by DCF addition as can be seen by the increase of $\Delta T_{1/2}$ values. The enthalpic variation ΔH associated to the main phase transition was almost unchanged by the presence of the drug. Together these observations confirm the shallower location of DCF at the C_1-C_8 region of the phospholipid acyl chains.

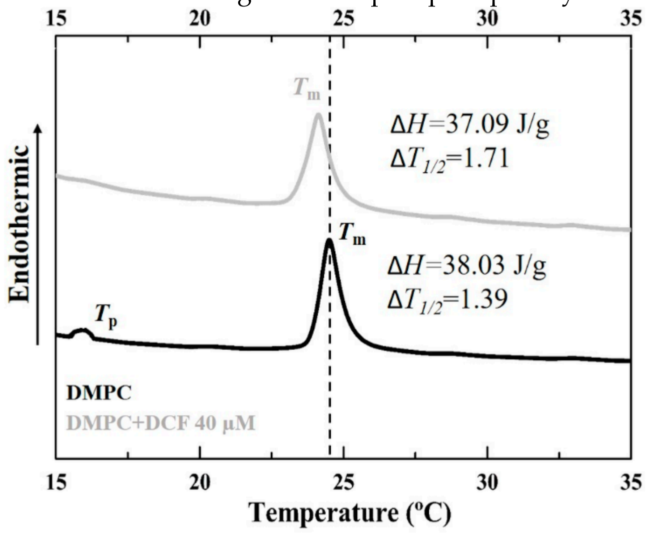

Figure S1. DSC thermograms of GI mimetic model systems at pH 5.0 in the absence and in the presence of DCF. The thermotropic parameters T_p, T_m, ΔH and $\Delta T_{1/2}$ stand respectively for: pre-transition temperature; main transition temperature; enthalpy variation associated to the main phase transition and cooperativity of the main phase transition.

Effects of Diclofenac in lipid packing and membrane structure

Synchrotron Small- and wide-angle X-ray diffraction studies (SAXS and WAXS) were performed to evaluate the interactions of DCF with a palmitoyl rich mimetic system (DPPC) of the GI lining protective layer. Therefore, the pH of analysis was again pH 5.0 for the abovementioned reasons. Diffraction patterns of DPPC multilayers in the presence of 40 μM of DCF were obtained using a temperature scan from 20 to 50 °C to analyze the effect of the NSAID in different lipid phases (Figure S2). Comparing to DPPC diffraction patterns previously published, the DPPC+DCF SAXS diffraction patterns (Figure S2A) originate broader and less intense Bragg peaks, sometimes presenting indications of phase separation, i.e. coexistence phases more and less influenced by the drug.

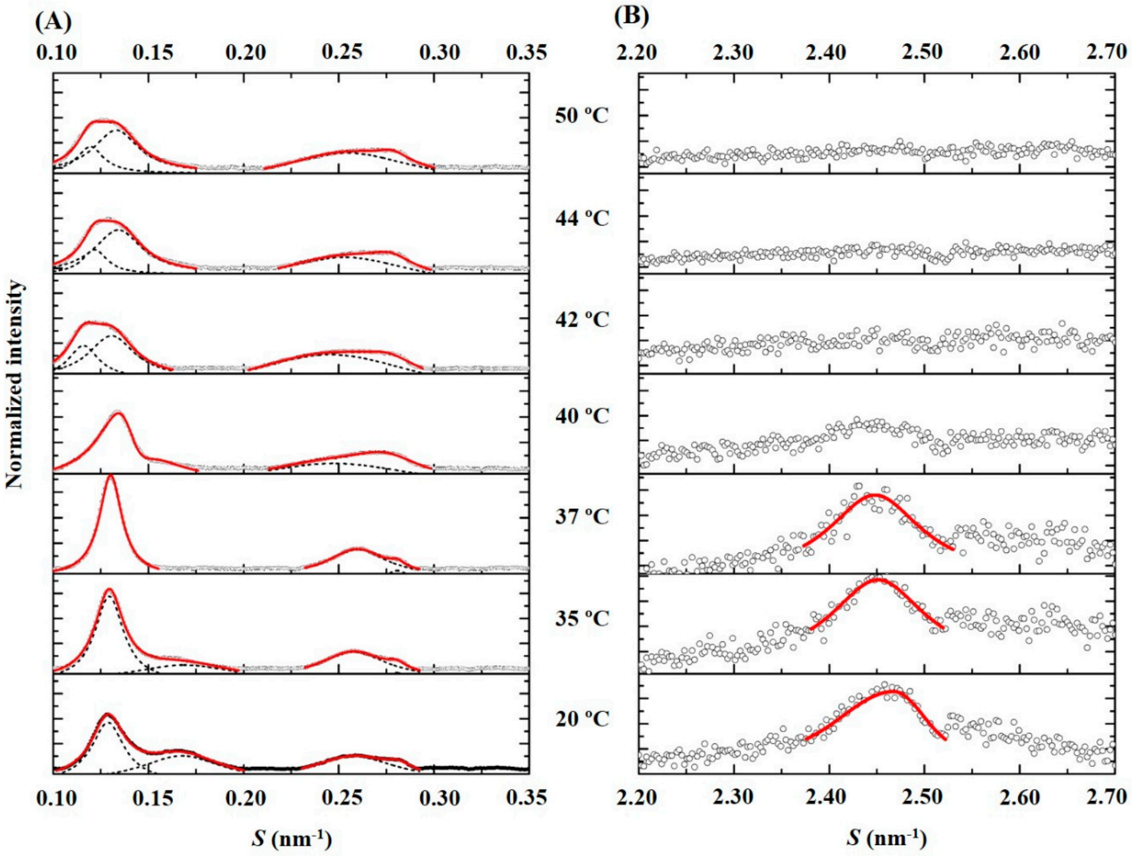

Figure S2. Temperature-dependent of X-ray diffraction patterns, SAXS (A) and WAXS (B) of DPPC containing DCF (40 μM) at pH 5. Solid red lines give the best fit of the Lorentzian's analysis model to the scattered intensities.

From the SAXS and WAXS patterns the spacings were calculated for each reflection order and the average value ± STD obtained for all the Bragg peaks are presented in Table S2.

Table S2. Long and short spacings determined from SAXS and WAXS patterns respectively of DPPC without or with DCF (40 μM) at different temperatures and at pH 5.0.

**Long spacings (SAXS)**						
**Temperature (°C)**	**Lamellar phase**		**Average *d* (Å)**		**ξ (Å)**	
	**DPPC**	**DPPC+DCF**	**DPPC**	**DPPC+DCF**	**DPPC**	**DPPC+DCF**
**20**	$L_{\beta'}$	L_{β}	65.4 ± 0.5	71.5 ± 1.0	592 ± 10	264 ± 10
**36**	$L_{\beta'}$	L_{β}	65.5 ± 0.5	71.3 ± 1.4	590 ± 10	304 ± 10
**38**	$P_{\beta'}$	L_{β}	66.7 ± 0.5	75.5 ± 0.9	580 ± 10	383 ± 10
**40**	$P_{\beta'}$	L_{α}	67.0 ± 0.5	75.6 ± 0.5	573 ± 10	199 ± 10
**42**	L_{α}	L_{α}	72.0 ± 0.5	78.3 ± 1.6	549 ± 10	288 ± 10
**44**	L_{α}	L_{α}	74.0 ± 0.5	76.9 ± 0.9	550 ± 10	344 ± 10
**50**	L_{α}	L_{α}	74.6 ± 0.5	77.3 ± 1.4	548± 10	326 ± 10

**Short spacings (WAXS)**						
**Temperature (°C)**	**d_{20} (Å)**		**d_{11} (Å)**		**A_0 (Å^2)**	
	**DPPC**	**DPPC+DCF**	**DPPC**	**DPPC+DCF**	**DPPC**	**DPPC+DCF**
**20**	4.17 ± 0.05	*	4.05 ± 0.05	4.07 ± 0.05	20.1	19.0
**36**	4.17 ± 0.05	*	4.05 ± 0.05	4.08 ± 0.05		
**38**	4.16 ± 0.05	*	*	4.08 ± 0.05		
**40**	4.15 ± 0.05	*	*	*		
**42**	*	*	*	*		
**44**	*	*	*	*		
**50**	*	*	*	*		

* represents absence of any Bragg peaks; $L_{\beta'}$, L_{β}, $P_{\beta'}$ and L_{α} stand respectively for lamellar gel tilted phase, lamellar gel untilted phase, ripple phase and lamellar fluid phase; A_0 is the cross-sectional area of an aliphatic chain calculated at 20 °C .
